# Superordinate identities and self-transcendent emotions: Longitudinal study in Spain and Chile

**DOI:** 10.3389/fpsyg.2022.989850

**Published:** 2022-11-11

**Authors:** Anna Wlodarczyk, Lander Méndez, Olaia Cusi, Saioa Telletxea, Jara Mendia, Mauricio Briceño, Daniela Delgado, Francisca Balbontín, Alexandra Lecaros, Darío Páez

**Affiliations:** ^1^Escuela de Psicología, Universidad Católica del Norte, Antofagasta, Chile; ^2^Department of Social Psychology, Faculty of Psychology, University of the Basque Country UPV/EHU, San Sebastián, Spain; ^3^Department of Basic Psychological Processes and Their Development, University of the Basque Country UPV/EHU, San Sebastian, Spain; ^4^PhD Programme in Education and Society, Faculty of Education and Social Sciences at Universidad Andrés Bello, Santiago de Chile, Chile

**Keywords:** superordinate identities, identification with all of humanity, self-transcendent emotions, self-oriented emotions, pandemic, COVID-19, IWAH

## Abstract

Recent studies suggest that identification with all humanity (IWAH), apart from being related to universalistic values, could also be related to self-transcendent emotions (STE). In this scenario, the general objective of this cross-cultural longitudinal study is to examine the relationship between identification with proximate categories (i.e., community and country) and superordinate one (all humanity), and their association with positive self-oriented and STEs during a traumatic global phenomenon such as COVID-19 pandemics. Additionally, we explore variations regarding the patterns of those associations in different cultural contexts (Chile and Spain) and examine whether they change among two different time points (T1–T2). The total sample was composed of 403 participants, of whom 224 were residents in Chile (*M* = 39.25, *SD* = 12.56; range 18–71 years; 49.6% women) and 179 were residents in Spain (*M* = 36.35, *SD* = 12.12; range 18–68 years; 59.8% women). Data collection was carried out in September (T1) and November (T2) 2020, through online surveys administered via Survey Monkey^®^ platform. Overall, results show, as expected, greater identification with proximate categories rather than superordinate ones, and an association between STEs and IWAH, but also with national and community identification. IWAH, but not STEs decreased significantly (T1–T2) in both countries. Thereafter, these emotional and behavioral responses decline as a symptom of growing fatigue with the pandemic situation, and also reflect a shift from broader to more local concerns. Analysis regarding comparisons between countries indicated higher levels of identification with community and with all humanity in Spain and with country in Chile. The results are discussed in the context of new developments in studies on IWAH.

## Introduction

The recent pandemic affected humans’ lives on an enormous scale, both at an individual and collective level. This experience showed all of us that more than ever we require common solutions for shared problems and collective threats. However, in the absence of a global governance system (Dietz, 2003; Held, 2010), it is necessary for individuals to develop capacities that enable them to respond individually or collectively to these challenges. According to Bartelson (2008), the greatest obstacle to the conceptualization of a global society is the division of humanity into groups, since these create identities that are possibly adversarial or in conflict. Therefore, a superordinate, inclusive identity is required, incorporating the diversity of current social distinctions, and encompassing the entire world. This could be the perception of being mutually linked with all humanity (Karlberg, 2008; McFarland et al., 2019). Although identification with all humanity (IWAH) has been studied mainly as a latent individual characteristic, which could be developed through socialization processes, we believe that it is important to investigate circumstances, conditions and mechanisms that could help to encourage, promote it or enlarge it (see Reysen et al., 2022; Sparkman et al., 2022). In this regard, the pandemic could promote inclusive attitudes by creating a common in-group identification in the face of a global threat, because psychosocial responses of the general population to previous pandemics included a greater sense of empowerment and compassion toward others (Taylor, 2019; Chew et al., 2020). This shows that not only in-group cohesion and out-group exclusion and stigmatization are triggered by an epidemic, but inclusion and solidarity can also occur (see Páez and Pérez, 2020). As de Rosa and Mannarini (2020) argue, collective traumatic events and threats, such as COVID-19, can increase awareness of human vulnerability, consequently creating broader representations of identity where the in-group is considered to be the humanity as a whole. On the other hand, recent studies have shown that IWAH, apart from being potentially triggered by collective threats, is also related to shared benevolence and universalistic values (Daniel et al., 2014; McFarland et al., 2019), and self-transcendent emotions (STE) (Alfaro-Beracoechea et al., 2019; Alfaro-Beracoechea and Contreras-Tinoco, 2021; Pizarro et al., 2021a). In fact, the induction of three different STEs, even controlling for individuals’ value orientations, explained fusion of identity with all humanity, as well as collective intentions to help others (Pizarro et al., 2021b). Nevertheless, as far as we know there are no recent studies which would analyze those links, STEs and superordinate identities, during these adverse pandemic times. Therefore, considering the challenges of the unpredictable scenario established by the COVID-19 pandemics, it is necessary to examine the levels of identification (proximate and superordinate ones) and to explore its relation with self-transcendent and self-oriented emotions (SOE) (and the associations between the two). Additional, in line with other studies carried out in the midst of the pandemic (Brooks et al., 2020; Ruiz et al., 2021), we believe it is important to emphasize the impact produced by quarantine, as well as the differences that can be found in the comparison between countries, since most of the studies are based only on single country samples (see for example: Bauer et al., 2020 in Germany; Camitan and Bajin, 2021 in Philippines; Enea et al., 2021 in Romania; Fawaz et al., 2021 in Lebanon; Filgueiras and Stults-Kolehmainen, 2020 in Brazil; Khan et al., 2020 in Bangladesh; Moreira et al., 2020 in Portugal; Mousavi, 2020 in Iran; Landi et al., 2020 in Italy; Liu et al., 2020 in China; López Steinmetz et al., 2022 in Argentina; Rodríguez-Rey et al., 2020 in Spain; Rosen et al., 2020 in the United States; van Tilburg et al., 2020 in the Netherlands). That is why we decided to focus on two different cultural regions (Spain and Chile) and explore the changes over time during the first wave of COVID-19 pandemic.

### Proximate identifications: Community and country

The Social Identity Theory (Tajfel and Turner, 1979) has been very useful in helping to understand the nature of psychological affiliation to large social groups such as community and nation. These types of proximate identification refer to a specific subtype of social identity (Smith et al., 2005; Nigbur and Cinnirella, 2007); the self-recognition and the active identification of individuals along with an emotional attachment or belonging to a nation or community (Der-Karabetian and Ruiz, 1997).

Social Identity Theory (Tajfel and Turner, 1979, 1986; Tajfel, 1981) states that individuals tend to make favorable differentiations for the in-group (“us”) and as a consequence behavior such as prejudice, discrimination, ethnocentrism, stereotypes and prejudices may arise in relation to the out-group, for example, to people from other countries or cultures (“them”). In addition, a strong and positive proximate identification (in-group: community and country) presupposes feelings of belonging, satisfaction and pride, as well as engagement and participation in one’s own social and cultural practices. In this sense, strong proximate identities may imply a reactionary position that, in the long run, can produce an incubation of contradictions and resentments that at a certain point might provoke unfavorable reactions toward the out-group (Stupar-Rutenfrans et al., 2021; Zhai and Yan, 2022), for example, to people from other countries or cultures. However, it also may represent an element of union and cohesion in the face of external aggression (from another country or the dominant culture against the minority culture or a natural catastrophe like COVID-19 pandemics). Studies show the importance of in-group social identity, as well as reaffirm that experiences of catastrophes induce inclusive helping behaviors. A study found that personal exposure to COVID-19 increases prosocial behavior (charitable donations), but this increased altruism is mostly in-group oriented: donors predominantly benefited the local level because donations toward country and world levels were half as large. Moreover, confirming the importance of social identity, the greater the identification with the local collective, the more donations for this in-group, but, at the same time, the greater the identification with the world, the more donations to the world in general (Grimalda et al., 2021).

In contrast to proximate identifications or in-group identifications, like identification with the world or superordinate identification categories implies seeing ourselves as members of the human race, regardless of nationality, culture, religion, race, political orientation, or socioeconomic status (McFarland et al., 2012, 2013). In this line, McFarland et al. (2012) used the concept of IWAH to define a type of social identity that refers to recognizing that all of humanity is one’s in-group.

### Superordinate identity: Identification with all humanity

The idea of IWAH dates back to the times of Diogenes of Sinope (412 BC) and Chrysippus who argued that cosmopolitanism would take hold by diminishing the importance of national identities. Within modern psychology, the concept of IWAH appeared in the theories of Adler, Maslow, Allport, and Erikson (for a more extensive review, see Hamer et al., 2019; McFarland et al., 2019). Contemporaneously, it is worth mentioning studies devoted to predictors and consequences of IWAH (McFarland et al., 2012; Hamer et al., 2017, 2018, 2019; McFarland, 2017), psychological sense of being part of a global community (Hackett et al., 2015), identification with the world at large (Buchan et al., 2011), the concept of global citizenship (Katzarska-Miller et al., 2012), and world citizenship (Türken and Rudmin, 2013).

By definition, it is assumed that IWAH refers to being aware of and willing to engage in combating global problems of the human race that concern us all (such as climate change or human rights violations) by adopting practices and values of a global culture (McFarland et al., 2012; Reese et al., 2015) such as democracy, human rights, and freedom. In terms of consequences, studies reveal that IWAH predicts human rights orientation, concern for global issues (McFarland et al., 2012, 2019), prosocial activities toward people from different countries, cultures intergroup forgiveness (Hamer et al., 2017, 2018), acceptance of refugees, as well as support for international volunteering and international charities (McFarland et al., 2019).

Furthermore, several studies confirmed that openness to experience, empathy, and universalism-tolerance values are the psychological underpinnings of this identification, whereas ethnocentrism, blind patriotism, right-wing authoritarianism, social dominance orientation, and religious fundamentalism are negatively related (Hamer et al., 2019; McFarland et al., 2019). However, the general pattern that has been found is that identification with the community, the nation, and all of humanity are positively associated and not contradictory (McFarland and Hornsby, 2015; Alfaro-Beracoechea et al., 2019). Although in general people identify more with the community or the nation as compared to superordinate levels such as all humanity (Hamer and Gutowski, 2009; McFarland et al., 2019; Hamer et al., 2021).

Nevertheless, studies on cross-cultural validation and replicability of these findings are still scarce, especially outside Europe and the United States. Consequently, as McFarland et al. (2019) point out, there is a need to test these results using more complex measures and to expand this research to different cultural contexts, especially underrepresented ones (a representative sample from Africa, Oceania, Latin America, rural areas, and small cities would be needed).

### Self-oriented and self-transcendent emotions

Within the various emotional families, a classic dichotomization of emotions is based on their orientation; SOE and others-oriented emotions. Fernández et al. (2020) point out the importance of distinguishing between them since they respond to different motivations and their connotation differs significantly. The former are focused on internal states and individual goals, while the latter pay more attention to the social context and the interdependence of others (Piff and Moskowitz, 2018). That is, in the case of SOE, the motivation is selfish, seeking to mitigate one’s own discomfort, while the motivation of others-oriented emotions is altruistic; they are evoked from a feeling of empathy toward the suffering of others and their final goal is to mitigate it (see Fernández et al., 2020). Therefore, STEs tend to reduce or minimize the importance of and attention to oneself, so they are close to others-oriented emotions (Fu et al., 2022). Additionally, numerous studies propose that STEs increase positive behaviors and attitudes toward different out-groups and foster social inclusion (see Van Cappellen and Saroglou, 2012; Yaden et al., 2017); hence, they may be related to higher levels of IWAH (de Rivera, 2018; McFarland et al., 2019).

Various studies proved that individuals experience STEs, such as kama muta or ‘being moved by love’ (Van Cappellen et al., 2013; Seibt et al., 2017, 2018; Zickfeld et al., 2017; Schubert et al., 2018; Fiske et al., 2019) or moral elevation (Haidt, 2000, 2003; Algoe and Haidt, 2009; Aquino et al., 2011; Diessner et al., 2013; Pohling and Diessner, 2016), while being exposed to touching or moving stimuli. In other words, the experiences that elicit these emotions are intrinsically social and have been described as involving issues of affiliation and social relationships, and evaluations of shared moral virtues, and they allow to elaborate a more complex understanding of others and increase proximity and overcoming obstacles (Shiota et al., 2007; Haidt and Morris, 2009; Rimé, 2009; Cova and Deonna, 2014; Menninghaus et al., 2015; Stellar et al., 2017).

Consequently, studies have found that STEs like elevation and social awe, can be evoked by exemplary actions of health care workers and by supportive community responses to the pandemic of COVID-19 (Páez and Pérez, 2020; Zlobina and Dávila, 2022). Thus, it is possible that the pandemic may have triggered these emotional reactions to poignant experiences such as, for example, health care workers giving their lives for the lives of others, stories of separation and reunion after ICU, stories about the death of the loved ones, suffering for others, acts of kindness, compassion, sacrifice, generosity, etc. That is, circumstances that can move and inspire individuals to experience a feeling of devotion to a common identity.

### Relationship between self-transcendent emotions and identification with all humanity

Within the family of positive emotions, STEs can mobilize individuals to connect with the people around them (Van Cappellen and Rimé, 2014). Some studies have previously shown that these emotions motivate the strengthening of more inclusive identities which can be translated into an increase in people’s sense of identification with humanity (Pizarro et al., 2021a,b).

For example, studies show that STEs such as kama muta make people feel love, solidarity, compassion and identification with others (Zickfeld et al., 2017; Pizarro et al., 2021b). That is, individuals feel oneness, union, or dissolution of the self into some bigger entity such as nature, earth, or the cosmos (Schubert et al., 2018; Seibt et al., 2018). On the other hand, Bai et al. (2017), in line with other studies (i.e., Shiota et al., 2007; Pizarro et al., 2021b) found that awe can reduce individual self-consciousness and self-absorption, thus promoting identification with the culture of the collective, for example, with one’s own community. Consequently, the experience of awe causes individuals’ egocentric tendencies to decrease and their collective identity to be strengthened, which ultimately turns people’s attention to the broader collective (Piff et al., 2015; Wijk, 2021). Specifically, studies show that by increasing the perception of the self as small, this emotion boosts global citizenship identification, understood as feelings of connectedness or oneness with the world (Seo et al., 2022).

Furthermore, Negami (2020) found that threat-based awe, due to its association with powerlessness, would facilitate engagement with smaller social groups, compared to positive awe, which would be related to superior groups. In line with these results, Wang et al. (2020) also showed that negative awe has a positive effect on enhancing national identity. Accordingly, a recent study has shown that STEs, especially awe and kama muta were evoked more strongly during small informal group meetings, than during large collective and societal experiences (Cusi et al., 2022). These results suggest that STEs are related to all forms of social identifications. Therefore, it is important to remember that proximate or in-group identifications are not contradictory with superordinate identification (McFarland and Hornsby, 2015; Alfaro-Beracoechea et al., 2019).

In general, it is known that the various manifestations of STEs like elevation, awe and kama muta have shown strong associations with values which emphasizes appreciation, protection and voluntary concern for the well-being of others (Cusi et al., 2018; Pizarro et al., 2018, 2021a,2021b; Negami, 2020) and prosocial behavior (Daniel et al., 2014; McFarland et al., 2019). In addition, they also show associations with identity fusion (Pizarro et al., 2021b), greater focus on social identity (Rufi et al., 2016) and disposition to celebrate and honor our common humanity (Cusi et al., 2018; Pizarro et al., 2018).

In sum, given that it has been shown that STEs can promote both proximate and superordinate identifications it is especially necessary to explore those associations especially in uncertain and difficult times.

### Current state of issue

In a two-time longitudinal design, we analyze the responses of the general population of Chile and Spain collected in September and November 2020, when each country was going through its particular sanitary situation.

In Spain, the alert declaration approved by Parliament on March 14, 2020, granted sole command to the central government to manage the health crisis. It brought a lockdown of the entire population and mobility restrictions until June 21, 2020, when the decree was lifted, and the autonomous governments took control over their respective Autonomous Communities. From then on, free movement within the national territory was allowed, although borders with other countries by land and air were not opened until July 1, 2020. After the lockdown in the summer period (July and August), new infections increased progressively and significantly, which constituted the so-called “second-wave” of coronavirus. In the first half of September, 122,700 new cases were recorded and the total number of deaths since the beginning of the pandemic reached 30,000. The second half ended with 778,607 people diagnosed positive for coronavirus, a 16.7% occupancy rate in Intensive Care Units (ICU) and a total of 31,973 deaths nationwide (Ministerio de Sanidad, 2020a). By November 2020 the total number of infected persons was 1,656,444, ICU occupancy reached 26.3% and the number of deceased persons reached 45,511 (Ministerio de Sanidad, 2020b). Given that data, regional governments began to implement restrictive measures relating to the closure of non-essential stores, and restaurants and implemented perimeter closures even between municipalities and basic health areas (Basque Country, Region of Murcia and Madrid).

In Chile, on March 3rd, Chilean health authorities reported the first case of COVID-19. Two weeks later, on March 16th, the government announced the closure of universities, schools and 2 days after, on March 18th, the closure of the country borders and declaration of the national emergency, accompanied by several concrete interventions to further contain the outbreak in the region (MINSAL, 2020). Soon after, on March 22nd, the government declared a night-time curfew, localized lockdowns (i.e., intermittent lockdowns at the municipality level depending on total cases and case growth) and the first total quarantine period, on March 25th (Ministerio de Salud, 2020a).

By July 19th, the Chilean government implemented the “step by step” strategy. This plan considered five different stages of gradual opening, at the municipality level, based on the monitoring of epidemiological and health system indicators. In September, Chile had a total of 462,991 infections, an ICU occupancy rate of 39% and a death toll of 12,741 people (Ministerio de Salud, 2020a), with partial lockdowns until the end of the month in most regions. In November, after declaring greater mobility freedoms for a large part of the country’s regions, there was an increase in the number of infected persons (552,864), in ICU occupancy (76.4%), and in the number of deaths, which amounted to 15,430 (Ministerio de Salud, 2020b). These rates demanded a new partial quarantine period which lasted until December 2020 in Santiago de Chile and other regions.

Studies on responses to collective disasters have found that in a first phase (called the honeymoon phase) there is a positive emotional arousal and a significant altruistic response and openness to those affected, which lasts for a few weeks. Thereafter, these emotional and behavioral responses decline, and people focus on more limited issues (Rimé, 2020). For instance, collective applause to health workers and helping to members of the community were important at the beginning of the pandemic and after some weeks declined (Zlobina and Dávila, 2022).

### Objectives and hypotheses

Despite the long theoretical research on IWAH, on the one hand, and STEs on the other, empirical evidence examining their relationship is almost non-existent. In this scenario, the present study aims to examine the pattern of relationships between different levels of identification and their association with STEs in two different countries in the context of the COVID-19 pandemic. Specifically, we first propose to test whether proximate identifications (i.e., community and country) are compatible with superordinate identifications (i.e., all humanity). Second, we will explore how these are associated with experiences of STEs. Hence, these are the hypotheses on which this study is based:

(H1a) We expect that the levels of proximate identifications (community and country) will be higher than the level of superordinate identification (IWAH). (H1b) We assume that the relationship between all the different levels of identification will be positive.

(H2a) STEs should be strongly associated with both proximate and superordinate identifications, more than SOE. (H2b) We also expect that the more STEs participants experience, the more they will identify with a superordinate category (IWAH).

(H3) In addition, we expect a decline in STEs and all humanity identification from Time 1 to Time 2.

(H4) Finally, as a more exploratory goal, we want to know if there are mean differences between the two countries, as well as differences in the association between the study variables (levels of identification and emotions).

## Materials and methods

### Participants and procedure

The total number of participants was 403 persons of legal age, of whom 224 were residents in Chile (*M* = 39.25, *SD* = 12.56; range 18–71 years; 49.6% women) and 179 were residents in Spain (*M* = 36.35, *SD* = 12.12; range 18–68 years; 59.8% women). Sociodemographic characteristics of the sample can be found in Supplementary Table 1. The questionnaires were administered through online surveys, created on the Survey Monkey^®^ platform. The data collection process took place between September and November 2020. We contracted the services of Offerwise, a research panel company that provides programming, data collection, processing and analysis services^1^. This company convenes panelists through television advertising and social networks, currently managing a panel of more than 6 million active participants, who have their socio-demographic characteristics mapped and classified into consumer profiles. In this way, it was possible to adjust the recruitment to the characteristics of the population required for the present research, i.e., Chilean and Spanish citizens over 18 years of age. Finally, all participants received informed consent for the study, in which the voluntariness and anonymity of their participation were made explicit. The procedure was approved by the Ethics Committee of the Universidad Católica del Norte (Ref: 0041/2019).

### Measures

Fredrickson’s Positivity Test (Fredrickson, 2009; Wlodarczyk et al., 2021). This scale examines various positive emotional reactions that may have been experienced due to the coronavirus outbreak. It was decided to divide positive emotions into two categories or emotional families: self-oriented and self-transcendent. In this sense, we calculated two indexes: first, SOE composed of the average of two items (”What is the most joyful, glad, or happy you felt?” and “What is the most serene, content, or peaceful you felt?”); and second, STE composed of the average of five items which are particularly related to self-transcendence (Emmons, 2005) (”What is the most awe, wonder, or amazement you felt?”; “What is the most grateful, appreciative, or thankful you felt?”; “What is the most hopeful, optimistic, or encouraged you felt?”; “What is the most inspired, uplifted, or elevated you felt?”; and “What is the most love, closeness, or trust you felt?”). Participants responded ranging from “Not at all” to “Extremely.”

Identification with all humanity (McFarland et al., 2012; Hamer et al., 2021). The full-scale captures in 27 items concern and supportive behavior toward the disadvantaged, an endorsement of human rights and strong responses in favor of community, country, and global harmony. Specifically, participants must answer whether they agree with each of the items (e.g., “How much would you say you have in common with the following groups?”) that make up this scale and answer according to each social or identity category (1 = “My Community”; 2 = “My Country” and; 3 = “All Humanity”), ranging from 1 (lowest score) to 5 (highest score). In order to carry out the analyses shown below, variables were created. For this purpose, the corresponding items were grouped by each of the social categories explored in this study (”My Community,” “My Country,” and “All Humanity”). Based on CFA presented in previous studies (please see Reese et al., 2015; Reysen and Hackett, 2016; Sparkman and Hamer, 2020; Hamer et al., 2021; Sparkman, 2022) we decided to calculate two subscales of the “All Humanity” dimension. First subscale is called “Bond” (items 1–4), it refers to the cognitive categorization and affective feelings of closeness linked to the group. Second one is called “Concern” (items 6–9), and it refers to the feelings of care and responsibility for people all over the world. Since item 5 loaded on both factors (as in the aforementioned previous study, see Hamer et al., 2021), we decided to exclude it from both subscales (the Spanish IWAH scale can be found in the Supplementary Appendix). Indicators of internal consistency showed acceptable scores for both scales (see Supplementary Table 2).

## Results

First, regarding H1a, in order to compare the levels of proximate (community and country) and superordinate (all humanity) identifications we carried out *t*-test analysis and calculated their effect size using Cohen’s *d* (Lovakov and Agadullina, 2021; see Table 1. In favor of what was hypothesized, participants scored higher on identification with their own community [*t*(392) = 12.513; *p* = 0.000; *d* = 0.613] and their country [*t*(393) = 5.636; *p* = 0.000; *d* = 0.239] compared to IWAH. In this sense, it is observed that participants reported higher identification with their own community, followed by their country, and finally, with humanity as a whole. However, the concern subscale of all humanity dimension showed higher mean scores than all other levels of identification, being these differences statistically significant in the case of identification with one’s own community [*t*(394) = –3.378; *p* = 0.001; *d* = 0.172], as well as in the case of identification with one’s own country [*t*(396) = –10.280; *p* = 0.000; *d* = 0.507].

**TABLE 1:** Means, standard deviations and correlations among IWAH scale’s dimensions and subscales in the total sample.

Variables	*M*	*SD*	Community	Country	Humanity	Bond
Community	3.54	0.69	–	–	–	–
Country	3.27	0.71	0.51	–	–	–
Humanity	3.11	0.70	0.52	0.62	–	–
Bond	2.57	0.77	0.42	0.59	0.87	–
Concern	3.67	0.81	0.48	0.51	0.88	0.54

*N* = 403.

The reported scores refer to the average of T1 and T2. Pearson correlation (unilateral).

All correlations are significant at *p* ≤ 0.001.

In addition, Pearson correlation analyses were carried out to explore the association among all levels of identification (see also Table 1). Supporting H1b, we obtained a significant positive association between proximate identifications (community and country) and superordinate identification (all humanity: bond and concern), with medium-high effect sizes.

Concerning H2a, STEs were related to both proximate and superordinate identifications as expected (see Table 2). However, partially at odds with H2b, STEs showed the strongest associations with the country dimension (*r* = 0.40), followed by all humanity dimension (*r* = 0.33), and finally, with the community dimension (*r* = 0.27). Considering that STEs are the central element of the study, we wanted to test the differences of the magnitude of the above presented correlations. For this purpose, we carried out sample-weighted correlations comparison analyses (Diedenhofen and Musch, 2015) taking as reference the *r*-value of the correlation between STEs and all humanity dimension (*r* = 0.33), and we compared it with the correlation values of the community (*r* = 0.27) and country (*r* = 0.40) dimensions, respectively. The comparison of correlations did not result statistically significant for community dimension (*z* = –1.300; *p* = 0.097), whereas for country dimension it did (*z* = 1.752; *p* = 0.040). Additionally, we performed partial correlations between self-oriented and STEs and IWAH controlling for identification with community and country (see Supplementary Table 3). Those correlations were not statistically significant in the whole sample, nevertheless when analyzed separately, in Spain correlation was positive for both emotions and in Chile only for self-transcendent ones (see Supplementary Table 3 for detailed analysis).

**TABLE 2:** Correlation comparison between emotional scales and IWAH scale’s dimensions and subscales in the total sample.

Variables	Community		Country		Humanity		Bond		Concern	
SOE	0.13^b^		0.19^a^		0.17^a^		0.26^a^		0.04	
STE	0.27^a^		0.40^a^		0.33^a^		0.38^a^		0.21^a^	

	***z***	***p***	***z***	***p***	***z***	***p***	***z***	***p***	***z***	***p***
	
	–3.529	0.002^b^	–5.457	0.000^a^	–4.101	0.000^a^	–3.153	0.001^a^	–4.234	0.000^a^

*N* = 403.

The reported scores refer to the average of T1 and T2.

Pearson correlation (unilateral).

^*a*^*p* ≤ 0.001; ^*b*^*p* ≤ 0.01.

To test group differences in correlations between countries we used Dunn and Clark’s (1969) *z*-statistic.

To explore whether there were any changes in the levels of the study variables from T1 to T2 in each country (H3), we carried out repeated measures analysis. Following what we expected, we observed a decline in the levels of STEs and IWAH from Time 1 to Time 2. As can be seen in Table 3, in the case of Spain and Chile, IWAH decreased, while all other variables remained stable. In Chile, participants reported lower identification with their country in T2. In both Chile and Spain, the mean score of the subscale of concern decreased significantly at T2, while the subscale of bond remained stable. Finally, respondents in Chile reported experimenting significantly more SOE in T2.

**TABLE 3:** Repeated measures analyses of the study variables between T1 and T2 by country.

Variables	Range	*M (SD)*		*F*	*Sig.*	η^2^
Spain		*T*_1_	*T*_2_			
Community	1–5	3.72 (0.68)	3.73 (0.72)	0.049	0.825	0.000
Country	1–5	3.20 (0.68)	3.13 (0.70)	3.229	0.074	0.018
Humanity	1–5	3.27 (0.61)	3.20 (0.70)	5.607	0.019^c^	0.031
Bond	1–5	2.50 (0.73)	2.51 (0.80)	0.117	0.732	0.001
Concern	1-5	4.01 (0.71)	3.89 (0.73)	11.211	0.001^a^	0.060
SOE	0–4	2.00 (0.94)	1.91 (0.93)	2.300	0.131	0.013
STE	0–4	2.04 (0.98)	1.96 (0.90)	1.380	0.242	0.008

**Chile**						

Community	1–5	3.41 (0.79)	3.39 (0.82)	0.155	0.694	0.001
Country	1–5	3.43 (0.80)	3.31 (0.83)	7.913	0.005^b^	0.035
Humanity	1–5	3.07 (0.83)	2.96 (0.84)	5.305	0.022^c^	0.024
Bond	1–5	2.64 (0.92)	2.62 (0.90)	0.141	0.708	0.001
Concern	1–5	3.57 (0.95)	3.35 (0.96)	14.957	0.001^a^	0.063
SOE	0–4	2.04 (1.01)	2.28 (1.14)	9.516	0.002^b^	0.041
STE	0–4	2.25 (0.96)	2.37 (1.06)	2.832	0.094	0.013

*n*_*Spain*_ = 179; *n*_*Chile*_ = 224.

^*a*^*p* ≤ 0.001; ^*b*^*p* ≤ 0.01; ^*c*^*p* ≤ 0.05.

Regarding differences between nations (H4), as can be seen in Table 4, there are statistically significant mean differences in all dimensions of the IWAH scale between Spain and Chile. Identification with community and all humanity mean scores were higher in Spain, while identification with the country was higher in Chile. Regarding the subscales identification of all humanity, Spanish residents reported higher levels of concern than Chilean ones, whereas the mean scores of bond subscale did not vary between the countries. Further, both self-oriented and STE’s mean scores were higher in Chile than in Spain.

**TABLE 4:** Means, standard deviations of the study variables, between-country *t*-test comparisons and effect sizes.

Variables	Spain *M (SD)*	Chile *M (SD)*	t-Test	Sig.	Cohen’s d
Community	3.72 (0.62)	3.40 (0.72)	4.664	0.000^a^	0.472
Country	3.16 (0.65)	3.37 (0.75)	–2.905	0.004^b^	0.297
Humanity	3.23 (0.62)	3.01 (0.75)	3.128	0.002^b^	0.316
Bond	2.50 (0.70)	2.62 (0.81)	–1.577	0.110	0.157
Concern	3.95 (0.67)	3.46 (0.85)	6.238	0.000^a^	0.632
SOE	1.95 (0.84)	2.16 (0.91)	–3.624	0.000^a^	0.239
STE	1.99 (0.84)	2.31 (0.86)	–2.326	0.020^c^	0.376

*n*_*Spain*_ = 179; *n*_*Chile*_ = 224.

The reported scores refer to the average of T1 and T2.

^*a*^*p* ≤ 0.001; ^*b*^*p* ≤ 0.01; ^*c*^*p* ≤ 0.05.

Furthermore, to explore possible differences between different cultural contexts characterized also by distinct health and mobility situations at the time of data collection (periods of non-strict confinement were inverse in Chile and Spain), we used a comparative *t*-test analysis to examine the differences between the two countries at both T1 and T2 separately (see Supplementary Table 4).

Results for both Spain and Chile show mean-above the arithmetic average at T1 (September), with higher levels in people residing in Spain compared to those who reside in Chile, on community dimension [*t*(395) = 4.086; *p* = 0.000; *d* = 0.424], all humanity dimension [*t*(395) = 2.712; *p* = 0.007; *d* = 0.271], and its concern subscale [*t*(398) = 5.206; *p* = 0.000; *d* = 0.518]. On the contrary, statistically higher means were found in Chile compared to Spain regarding country dimension [*t*(397) = –3.111; *p* = 0.002; *d* = 0.321] and STEs [*t*(397) = –2.240; *p* = 0.026; *d* = 0.217], although mean differences are not significant in the case of bond subscale [*t*(398) = –1.660; *p* = 0.098; *d* = 0.167], and SOE [*t*(397) = –0.428; *p* = 0.669; *d* = 0.041].

With respect to the differences found at T2 (November) comparing the Spanish and Chilean populations, it should be noted that the means were statistically higher in the case of community dimension [*t*(401) = 4.329; *p* = 0.000; *d* = 0.435], all humanity dimension [*t*(401) = 2.923; *p* = 0.004; *d* = 0.298], and concern subscale [*t*(401) = 6.173; *p* = 0.000; *d* = 0.625], with higher averages in the Spanish population. However, the Chilean sample showed statistically higher means of country dimension [*t*(401) = –2.465; *p* = 0.014; *d* = 0.246], SOE [*t*(401) = –3.468; *p* = 0.001; *d* = 0.353], and STEs [*t*(401) = –4.223; *p* = 0.000; *d* = 0.414]. As can be seen, the differences are not statistically significant in the case of bond subscale (*p* = 0.195), showing a similar pattern as at T1. The differences at T2 are not statistically significant in the case of bond subscale [*t*(401) = –1.299; *p* = 0.195; *d* = 0.129] as at T1. However, in contrast to T1, SOE did show an increase at T2.

To find out which country had undergone the greatest change over time in the study variables, we calculated the subtraction of T2 and T1 to obtain the resulting score as a comparative value. Cross-country mean-comparison analyses were carried out (see Table 5). The only differences found between Spain and Chile over time were in SOE (*d* = 0.318) and STEs (*d* = 0.193). No statistically significant differences were found in any of the dimensions nor subscales (bond and concern).

**TABLE 5:** Means, standard deviations, and t-test among the study variables by country and between time points (T2-T1).

Variables	Spain *M (SD)*	Chile *M (SD)*	t-Test	Sig.	Cohen’s d
Community	0.01 (0.64)	–0.01 (0.70)	0.430	0.668	0.030
Country	–0.06 (0.47)	–0.11 (0.61)	0.961	0.337	0.091
Humanity	–0.07 (0.42)	–0.11 (0.71)	0.570	0.569	0.067
Bond	0.01 (0.60)	–0.02 (0.80)	0.492	0.623	0.042
Concern	-0.12 (0.51)	–0.22 (0.84)	1.253	0.211	0.140
SOE	–0.09 (0.81)	0.23 (1.14)	–3.235	0.001^a^	0.318
STE	–0.07 (0.83)	0.11 (1.01)	–1.995	0.047^b^	0.193

*n*_*Spain*_ = 179; *n*_*Chile*_ = 224.

The reported mean-scores refer to the subtraction between T2 and T1.

^*a*^*p* ≤ 0.001; ^*b*^*p* ≤ 0.05.

Finally, to find out the association between the study variables in both countries, correlation analyses were carried out by summing both times, T1 and T2. As can be observed in Table 6, overall, correlations were stronger in Chile than in Spain, especially among different levels of identification (proximate as well as superordinate: community, country, and all humanity).

**TABLE 6:** Correlations of the study variables by country.

Variables	Community	Country	Humanity	Bond	Concern	SOE	STE
		**Spain**					
Community	–	0.43^a^	0.41^a^	0.34^a^	0.37^a^	0.02	0.30^a^
Country	0.64^a^	–	0.55^a^	0.44^a^	0.53^a^	0.10^d^	0.37^a^
Humanity	0.55^a^	0.72^a^	–	0.89^a^	0.88^a^	0.15^c^	0.34^c^
Bond	0.52^c^	0.66^c^	0.89^c^	–	0.58^c^	0.22^b^	0.34^c^
Concern	0.48^c^	0.62^c^	0.89^c^	0.60^c^	–	0.06	0.27^c^
SOE	0.25^c^	0.22^c^	0.21^c^	0.28^c^	0.09^d^	–	0.56^c^
STE	0.35^c^	0.40^c^	0.38^c^	0.40^c^	0.29^c^	0.74^c^	–
	
	Chile						

*n*_*Spain*_ = 179; *n*_*Chile*_ = 224.

The reported scores refer to the average of T1 and T2. Pearson correlation (unilateral).

^*a*^*p* ≤ 0.001; ^*b*^*p* ≤ 0.01; ^*c*^*p* ≤ 0.05; ^*d*^*p* ≤ 0.10.

Correlations above the diagonal correspond to the Spanish sample and those below the diagonal correspond to the Chilean sample.

Exploring the differences between Spain and Chile, between-country correlation analysis (Diedenhofen and Musch, 2015) showed statistically significant differences regarding associations between (a) community and country identification, (b) community and all humanity identification (also bound subscale), (c) country and all humanity identification (also bound subscale), and (d) SOE and community identification, being those associations higher in Chile (see Table 7).

**TABLE 7:** Between-country comparison of correlations among the study variables.

Variables	Community		Country		Humanity		Bond		Concern		SOE	
	*z*	*p*	*z*	*p*	*z*	*p*	*z*	*p*	*z*	*p*	*z*	*p*
Community	–	–	–	–	–	–	–	–	–	–	–	–
Country	3.027	0.001^a^	–	–	–	–	–	–	–	–	–	–
Humanity	1.845	0.033^c^	2.875	0.002^b^	–	–	–	–	–	–	–	–
Bond	2.216	0.013^c^	3.211	0.001^a^	0.049	0.481	–	–	–	–	–	–
Concern	1.309	0.095	1.210	0.112	0.602	0.274	0.275	0.392	–	–	–	–
SOE	–2.341	0.010^b^	–1.262	0.103	–0.675	0.250	–0.654	0.257	–0.349	0.364	–	–
STE	–0.587	0.279	–0.325	0.373	–0.434	0.332	–0.653	0.257	–0.279	0.390	–3.196	0.001^a^

*n*_*Spain*_ = 179; *n*_*Chile*_ = 224. The reported scores refer to the average of T1 and T2.

^*a*^*p* ≤ 0.001; ^*b*^*p* ≤ 0.01; ^*c*^*p* ≤ 0.05.

To test group differences in correlations between countries we used Dunn and Clark’s (1969) *z*-statistic.

Moreover, we conducted correlation analysis between the two families of emotions (self-oriented and self-transcendent) and the different levels of identification in T1 and T2 in Spain and Chile (see Supplementary Table 5). Overall, results show a greater association of STEs versus SOE with each level of identification in both times, and in both countries. In Spain, SOE were significantly related to all humanity dimension, yet only represented by bond subscale. On the other hand, STEs showed a positive and statistically significant association with all dimensions except for community dimension in T2. In Chile, all the associations were positive and statistically significant except for the relationship between all humanity dimension in T1 and SOE in T2.

In addition, Supplementary Table 6 shows the association between variables based on the mean correlation weighted by sample size. Overall, it shows the strongest associations of STEs with all levels of identification with medium effect size, evidencing the robustness of our results. Statistically significant *r*-values had a confidence interval that did not include zero.

## Discussion

The aim of this study was to explore the associations between STEs and proximate and superordinate identifications based on analysis of cross-cultural longitudinal data gathered during first wave of COVID-19 in 2020. Moreover, we sought to explore the existence of variations regarding the patterns of those relations in two different cultural contexts, that is in Chile and in Spain. Subsequently, we aimed at examining within-person and between country changes in the levels of proximate and superordinate identifications and emotions during two time points of the pandemic.

As to H1, our study partially confirmed what was expected, since the results showed that, in times of pandemic, participants reported greater identification with proximate identities. Specifically, they were more likely to identify with their own community, secondly with their country and finally with broader identifications such as all humanity. More precisely, it was concern (feelings of care and responsibility for people all over the world), the subscale of the all humanity dimension, which obtained the highest scores. In addition, in the total sample, all of the dimensions of the IWAH scale were positively associated with each other, indicating that a proximate level of identification does not exclude being able to identify oneself with superordinate categories (H1b).

Although both self-transcendent and SOE were associated with all the levels of identification, the former showed stronger associations with superordinate levels of identification, being all the differences statistically significant and supporting H2a. Globally, results confirmed that STEs are more strongly associated with different dimensions of identifications than SOE. Regarding H2b, STEs showed an association with all humanity dimension, but the strongest association was the one with country dimension.

Considering the longitudinal nature of our study, we wanted to explore the differences between T1 and T2 on the study variables (H3). In both countries, IWAH, but not STE levels decrease. In the case of Chile, the dimension of country decreased significantly. In contrast, SOE increased significantly in T2; this variable was the only one with a significant magnitude of change between T2 and T1 and between the countries. The results confirm that over time the initial inclusive response triggered by the collective catastrophe weakens (Rimé, 2020). However, the STEs are maintained, suggesting that these affective responses are more stable than the socio-cognitive ones of identification with the collective. This is an important and encouraging result, indicating that people find ways to generate emotions of connection with others despite isolation and a decline in initial altruistic responses.

With respect to between-country exploratory analysis (H4), we observed statistically significant differences regarding almost all the study variables in both countries. In the case of Spain, participants reported higher levels of identification with the community (this dimension being the one with the highest means) followed by IWAH, and its subscale of concern. The differences found between the means of subscale of bond, in comparison with Chile, were not statistically significant. On the other hand, Chile showed higher means for the country dimension. With respect to emotions, participants in Chile reported experimenting significantly more self-oriented and STEs in comparison with those form Spain. As for the associations between the variables, we found, in a generalized way, significantly greater strength between the study variables in Chile, both between the levels of identification and emotions.

COVID-19 has caused a great alteration in the way we live, feel and identify with social groups. In the psychological impact of the pandemic, it is difficult to identify aspects that have not been negatively marked at both the individual (Pedrosa et al., 2020) and group levels (Marmarosh et al., 2020). During the first wave of COVID-19, the enforced social isolation caused a distancing among individuals resulting in a decrease in social connectivity or affiliation (Best et al., 2020; Folk et al., 2020). Moreover, as the uncertainty spread, and mortality rates of the virus continued to increase during the second wave of data collection, individuals have been able to experience a need for survival (self-survival and survival of the people who are dearest or closest) that has diminished the ability to redirect their attention to the external, focusing on a specific more local area. Given that all decisions made at the local level directly affected our daily lives (e.g., availability of artificial ventilators, ICU beds, and healthcare workers, economic crisis, unemployment levels, among others), people were more likely to report concern for the local than for the superordinate community (see Abrams et al., 2021). That is, stressful and demanding conditions imply focusing on immediate need and a more concrete way of thinking (Caballero et al., 2021), which in consequence may hinder identification with broader categories.

In the case of our study, identification with the community was also more salient in both countries (particularly in Spain, where regional or ethnic identities like Basque or Catalonian are important) which could be explained by the tendency of human beings to identify more closely with proximate identities, with the pandemic being a factor that may have accentuated this tendency (see Wang et al., 2020). However, we also know that people can incorporate several levels of identity at the same time. A universally inclusive superordinate identity does not preclude the possibility of multiple and overlapping identities, whether ethnic, cultural, national or religious in origin, being grouped into a whole. As Karlberg (2008) put it, “A global ‘we’ can accommodate multiple secondary distinctions between ‘us’ and ‘them’ when those distinctions are not understood in a hostile or adversarial manner” (p. 315). An illustration of this view might be the integration strategy of Berry’s (1995) acculturation model, and the bicultural identity model of Benet-Martínez et al. (2002), according to which different cultural identifications can develop in parallel, and thus, to some extent, cease to be incompatible. Accordingly, our data show that all three levels of identification show positive associations, results that are also supported by other previous studies using the IWAH scale (see Hamer and Gutowski, 2009; Alfaro-Beracoechea et al., 2019; McFarland et al., 2019; Hamer et al., 2021; Sparkman, 2022; Sparkman et al., 2022).

Furthermore, our results considering country-comparison revealed that IWAH was higher among participants in Spain than in Chile. One of the possible explanations could be related to the differences in levels of national identification and patriotism (see Ariely, 2012; based on WVS data). Chilean participants reported higher levels of identification with the country, patriotism, and willingness to fight for it, as compared to the Spanish ones (Ariely, 2012). On the other hand, this result could be explained by the differences in religiosity levels between participants^2^ from both countries. As found by McCutcheon et al. (2015), IWAH was higher among skeptics than religious believers. Although Katzarska-Miller et al. (2014) did not find a significant association between global citizenship identification (superordinate) and religiosity. Therefore, it is evident that the cross-cultural literature concerning levels of IWAH and its predictors is not yet sufficiently consistent and rather scarce, thus, much more research is needed in this area (especially in the context of pandemics).

Accordingly, emotional mechanisms involved in enhancing broader social identifications in case of adverse circumstances are certainly complex and urgently require attention and rigorous research due to its important social implications. Thus, this study intended to examine positive emotions that may have some important influence in shaping the superordinate identifications. Several studies have confirmed the increase in negative emotions, stress, anxiety, and depression disorders, both, in Spain (Odriozola-González et al., 2020) and in Chile (Caqueo-Urízar et al., 2020). Our study focused partly on understanding the role of both self-oriented and STEs in times of pandemic.

Our results indicated that participants experienced more self-transcendent (than self-oriented) emotions. This is in line with studies on STEs that were conducted during collective traumatic events (Piff et al., 2015; Gordon et al., 2017; Septianto et al., 2022; Wang et al., 2022). For example, awe can be considered ambivalent since it can be triggered by both purely positive elements and by those that may be considered negative. In fact, awe is characterized as a self-diminish emotion, allowing individuals to orient themselves toward broader identities (Keltner and Haidt, 2003). Gordon et al. (2017) examined negative awe in threatening circumstances (e.g., tornadoes, terrorist attacks, angry God). Through a series of studies, they demonstrated that this STE can be evoked by threatening stimuli and that through eliciting the sense of a small self that characterizes it (see also Piff et al., 2015) individuals tend to create a sense of global community among people that ultimately increases participant’s willingness to help people in disaster-affected areas. In this regard, other studies also support these findings (Septianto et al., 2022; Wang et al., 2022), showing that when faced with powerful and even destructive catastrophes, people may be more likely to maintain a sense of smallness as part of the global community, paying less attention to their differences in race, nationality, and religion, reminding people of their common identity of a shared global community and promoting mutual aid.

Accordingly, recent studies also suggest that cultivating STEs serve as a buffer against pandemic suffering and help to minimize its negative impact on mental health (Wong et al., 2021). For example, an experimental study carried out by Datu et al. (2022) and held in the Philippines, highlights the importance of exploring STEs such as gratitude and kindness in times of crisis such as the pandemic, given the emotional benefits associated with the promotion of these emotions. Specifically, these authors found that these STEs (compared to the control condition) were associated with greater affective and emotional well-being, and they argued that cultivating these positive virtues may facilitate satisfaction of basic psychological needs. These results were explained by the fact that people in the gratitude and kindness condition showed significantly higher means of positive emotions than those in the control condition. Likewise, Algoe and Haidt (2009) show that STEs such as elevation, gratitude, and admiration differ from each other and, more importantly, are different from more commonly studied forms of positive affect such as joy and amusement.

Our data indicated a positive and strong relationship between self-transcendent and SOE. However, the STEs showed the strongest associations with all the social or collective identification levels. These results may suggest that, although related, these two types of emotions may involve qualitatively different effects, being the identification with collectives (local, national, and global) a central aspect of STEs. Moreover, our data demonstrated that STEs, as compared to self-oriented ones, were significantly more strongly associated with IWAH dimension. In short, according to other studies, we corroborated that self-transcendent and not SOE, allow individuals to connect with people and feel part of something bigger than the self, such as humanity (see Van Cappellen and Rimé, 2014; Pizarro et al., 2021a,b).

Regarding differences between countries, as to proximate identities, in Chile we observed a decrease in identification with the country (in Spain it remained stable). It may be related to citizens’ dissatisfaction with governments’ facing the COVID-19 pandemic as suggested by Hamer et al. (2021). Thus, lower satisfaction with the management of the pandemic by the authorities could be related to the decline in the identification with the country. In the case of IWAH, it turned out that between September and November of 2020, its levels decreased in both countries. Specifically, the aspect related to concern for people around the world declined. This decrease, which can be observed in both countries, may be considered another symptom of fatigue with the pandemic situation and was also found in other studies, for example, Hamer et al. (2021). On the other hand, the rise of SOE levels in Chile, may be influenced by socio-sanitary situation (i.e., lockdown). In November, the strict lockdown measures were lifted across various regions, which could be one of the reasons to experience more joy and serenity among the residents of the country.

It is important to note that understanding and studying self-transcendent positive emotions can help to understand the new gap that has been created within the field of positive and social psychology. In this respect, in recent years, emotional processes have become of interest to researchers in the framework of studies on relationships where social identity becomes salient. Such a conception, if well channeled, could help to develop an active change in the way individuals and specific social groups think (i.e., by defining themselves as members of the human race), and, perhaps, in how they act. On the other hand, the study of superordinate identities may also be relevant. According to the Common In-group Identity Model (Gaertner and Dovidio, 2000), the salience of a more inclusive superordinate identity category containing both in- and out-group identities reduces intergroup stereotypes and can foster harmony between conflicting groups (Gaertner and Dovidio, 2000; González and Brown, 2002, 2006; González et al., 2008).

Previous studies in the midst of the pandemic have already shown that individuals have diminished social cohesion and identification with broader categories feeding polarization and extremism, such as an increase in rejection toward the out-group, greater prejudice, discrimination, stigma, race-based threat, and xenophobia (Roberto et al., 2020; see Gover et al., 2020; Inman et al., 2021; Li and Nicholson, 2021; Gao, 2022; Kahn and Money, 2022). In this regard, the broadest, and at the same time the most inclusive, superordinate level of identification which is IWAH can help individuals, at least to some extent, reduce intergroup threats (Riek et al., 2010). Based on the common group identity model (e.g., Gaertner et al., 1993), if we want to reduce the intergroup bias, we need both groups to be recategorized as a single one. In this way, we can use tools such as STEs and superordinate categories as they lead us to connect with all of humanity (McFarland et al., 2012). Thus, maybe categorizing ourselves as just another citizen of the world could help to expand the mind and understand that we live interconnected, and what happens on the other side of the world can have serious repercussions on our countries, our people and our daily lives.

### Study limitations

Although our study makes several important contributions to the literature, of course it is not without its limitations. First, it should be noted that the samples used for the study were not entirely representative at the country level. On the other hand, despite the fact that most of the patterns of the results obtained in the Chilean context have also been confirmed in the Spanish, which definitely gave stability and scope to the results and conclusions of the study. Nevertheless, in order to better understand the relationships raised, it would be of great interest to expand the sample to other cultural contexts and countries as suggested by McFarland et al. (2019).

Another important limitation to consider is that, although one of the objectives of the study was to evaluate the relationship between IWAH and STEs in the context of a pandemic, this study has no precedents, so there is no empirical evidence on this matter. As a result of the aforementioned, it is not possible to make an adequate prediction of the behavior that people will maintain and how this will influence their IWAH. This is also related to the fact that the hypotheses proposed in the present study were not fulfilled, since, for example, it was expected that within the pandemic context to which the individuals were exposed, they would experience more SOE, whereas participants reported more STEs. All these limitations will be considered, and an attempt will be made to reduce them in future studies.

### Future research

Further studies are needed that include more representative population groups of different socio-economic and educational levels, ethnicity, etc., which would allow us to assess whether there are socio-demographic or socio-economic, or other psychosocial variables that could explain the differences in the interaction between these phenomena.

On the other hand, as Fredrickson (2002) suggests, there is a need for methods that make it possible to experience positive feelings more frequently in difficult moments of life, to keep an open mind, and to be more cooperative (Fredrickson, 2004). In times of COVID-19, it is essential to find ways to experience positive emotions that help us to decentralize our attention from the current shocking events for at least a moment. It is also necessary to have the concrete tools to try to experience these kinds of emotions that dislodge the self-focus and shift our attention away from the self and toward broader concepts.

Following this line, the study of the different STEs would also make it possible to design useful interventions for everyday life that not only seek to alleviate negative emotions but also produce positive effects on creating a superordinate identity.

## Data availability statement

The original contributions presented in this study are included in the article/Supplementary material, further inquiries can be directed to the corresponding authors.

## Ethics statement

The studies involving human participants were reviewed and approved by Ethics Committee of the Universidad Católica del Norte (Ref: 0041/2019). The patients/participants provided their written informed consent to participate in this study.

## Author contributions

AW was the primary investigator of the study, provided comments and ideas, and drafted and revised the manuscript. AW, LM, and OC worked equally in the data collection, data analysis performance and development, and revision of the manuscript. ST, JM, and DP contributed providing ideas and discussing the results. JM, MB, DD, FB, and AL contributed significantly to data collection. All authors contributed to the last revision and approved the submitted version of the manuscript.

## Footnotes

1
https://www.offerwise.com/

2 Participants from the Chilean sample reported significantly higher levels of religiosity [*t*(401) = –10,853; *p* = 0.000; see Supplementary Table 1].
