# Supplementary material for: Superordinate identities and self-transcendent emotions: Longitudinal study in Spain and Chile
Source: Front Psychol. 2022 Nov 11;13:989850. doi: 10.3389/fpsyg.2022.989850 (PMC9692013; doi:10.3389/fpsyg.2022.989850)
Supplement: Supplementary file 1 [file Table_1.docx]

***Supplementary Material***

# Supplementary Figures and Tables

**Supplementary Table 1**

*Sociodemographics by Country*

|  | | Minimum – Maximum | M (SD) | Frequency (%) |
| --- | --- | --- | --- | --- |
| **Spain** | Religiosity | 1-4 | 1.26 (.62) | - |
|  | Number of Children | 0-4 | 0.56 (.95) | - |
|  | Social Status | 5.5 – 80.5 | 49.10 (14.90) | - |
|  | Age | 18 - 68 | 36.44 (12.13) | - |
|  | Gender (Women) | - | - | 59.8 |
|  | Education |  |  |  |
|  | Elementary | - | - | 0.6 |
|  | High School | - | - | 6.7 |
|  | Undergraduate/Bachelor's Degree | - | - | 41.3 |
|  | Master's Degree | - | - | 30.2 |
|  | Doctorate | - | - | 16.2 |
|  | Still studying | - | - | 5.0 |
|  | No studies | - | - | 0 |
|  | Marital status |  |  |  |
|  | Single | - | - | 39.7 |
|  | Relationship | - | - | 28.5 |
|  | Married | - | - | 24.6 |
|  | Widowed | - | - | 1.1 |
|  | Divorced | - | - | 6.1 |
| **Chile** | Religiosity | 1-4 | 2.27 (1.11) | - |
|  | Number of Children | 0-7 | 1.24 (1.25) | - |
|  | Social Status | 3.5 – 97.5 | 49.51 (17.99) | - |
|  | Age | 18-71 | 39.36 (12.54) | - |
|  | Gender (Women) | - | - | 49.6 |
|  | Education |  |  |  |
|  | Elementary | - | - | 0.9 |
|  | High School | - | - | 21.4 |
|  | Undergraduate/Bachelor's Degree | - | - | 58.5 |
|  | Master's Degree | - | - | 7.6 |
|  | Doctorate | - | - | 1.3 |
|  | Still studying | - | - | 10.3 |
|  | No studies | - | - | 0 |
|  | Marital status |  |  |  |
|  | Single | - | - | 41.5 |
|  | Relationship | - | - | 12.5 |
|  | Married | - | - | 40.2 |
|  | Widowed | - | - | 0.4 |
|  | Divorced | - | - | 5.4 |

*Note: n_Spain_* = 179; *n_Chile_* = 224. Totals of T1 + T2 were created to draw means and frequencies. In the case of education and marital status, T2 was taken into account.
