# Supplementary material for: Superordinate identities and self-transcendent emotions: Longitudinal study in Spain and Chile
Source: Front Psychol. 2022 Nov 11;13:989850. doi: 10.3389/fpsyg.2022.989850 (PMC9692013; doi:10.3389/fpsyg.2022.989850)
Supplement: Supplementary file 2 [file Table_2.docx]

Supplementary Material

# Supplementary Figures and Tables

**Supplementary Table 2**

*Internal Consistency of the Study Variables by Country and by Time*

| **Variables** | **Items** | **Total T1 *α*** | **Total T2 *α*** | **Spain T1 *α*** | **Chile T1 *α*** | **Spain T2 *α*** | **Chile T2 *α*** |
| --- | --- | --- | --- | --- | --- | --- | --- |
| **Community** | 9 | .87 | .90 | .84 | .89 | .87 | .91 |
| **Country** | 9 | .86 | .88 | .85 | .88 | .85 | .90 |
| **Humanity** | 9 | .87 | .88 | .82 | .90 | .87 | .90 |
| **. Bond** | 4 | .80 | .83 | .73 | .84 | .82 | .84 |
| **. Concern** | 4 | .84 | .83 | .72 | .87 | .72 | .86 |
| **SOE** | 2 | .70 | .72 | .65 | .74 | .63 | .75 |
| **STE** | 5 | .80 | .81 | .76 | .83 | .75 | .84 |

*Note. N _=_* 403; *n_Spain_* = 179; *n_Chile_* = 224. α = Cronbach alpha.
