# Supplementary material for: Superordinate identities and self-transcendent emotions: Longitudinal study in Spain and Chile
Source: Front Psychol. 2022 Nov 11;13:989850. doi: 10.3389/fpsyg.2022.989850 (PMC9692013; doi:10.3389/fpsyg.2022.989850)
Supplement: Supplementary file 3 [file Table_3.docx]

***Supplementary Material***

# Supplementary Figures and Tables

**Supplementary Table 3**

*Partial Correlations of Self-oriented and Self-transcendent Emotions with All Humanity Dimension and its Subscales Controlling for Community and Country Dimensions by Country*

| **Variables** | **Humanity** | | **Bond** | | **Concern** | |
| --- | --- | --- | --- | --- | --- | --- |
| **Total Sample** | **-** | | **-** | | **-** | |
| **SOE** | .031 | | .161^a^ | | -.095 | |
| **STE** | .070 | | .170^a^ | | -.038 | |
|  | **z** | **p** | **z** | **p** | **z** | **p** |
|  | -0.961 | 0.336 | -0.225 | 0.821 | -1.407 | 0.159 |
| **Spain** | - | | - | | - | |
| **SOE** | .123^d^ | | .196^b^ | | .012 | |
| **STE** | .145^c^ | | .186^b^ | | .052 | |
|  | **z** | **p** | **z** | **p** | **z** | **p** |
|  | -0.314 | 0.753 | 0.144 | 0.885 | -0.566 | 0.571 |
| **Chile** | - | | - | | - | |
| **SOE** | .001 | | .119^c^ | | -.115^c^ | |
| **STE** | .104^d^ | | .156^c^ | | .020 | |
|  | **z** | **p** | **z** | **p** | **z** | **p** |
|  | -2.131 | 0.033^c^ | -0.771 | 0.440 | -2.796 | 0.005^b^ |

*Note.* *N* = 403. The reported scores refer to the average of T1 and T2. Pearson correlation (unilateral). ^a^*p* ≤ .001; ^b^*p* ≤ .01; ^c^*p* ≤ .05; ^d^*p* ≤ .10. We used z-statistic of Dunn & Clark (1969) to test group differences in correlations between SOE and STE.
