# Supplementary material for: Superordinate identities and self-transcendent emotions: Longitudinal study in Spain and Chile
Source: Front Psychol. 2022 Nov 11;13:989850. doi: 10.3389/fpsyg.2022.989850 (PMC9692013; doi:10.3389/fpsyg.2022.989850)
Supplement: Supplementary file 4 [file Table_4.docx]

***Supplementary Material***

# Supplementary Figures and Tables

**Supplementary Table 4**

*Means, Standard Deviations of the Study Variables (T1 & T2) and t-Test analysis by Country*

| **Variables** | **Spain**  ***M (DT)*** | **Chile**  ***M (DT)*** | ***t-Test*** | ***Sig.*** | ***Cohen's d*** |
| --- | --- | --- | --- | --- | --- |
| **Community T1** | 3.72 (0.67) | 3.41 (0.78) | 4.086 | .000^a^ | .424 |
| **Community T2** | 3.73 (0.72) | 3.39 (0.83) | 4.329 | .000^a^ | .435 |
| **Country T1** | 3.19 (0.68) | 3.43 (0.80) | -3.111 | .002^b^ | .321 |
| **Country T2** | 3.13 (0.70) | 3.32 (0.83) | -2.465 | .014^c^ | .246 |
| **Humanity T1** | 3.27 (0.61) | 3.07 (0.83) | 2.712 | .007^b^ | .271 |
| **Humanity T2** | 3.19 (0.70) | 2.96 (0.83) | 2.923 | .004^b^ | .298 |
| **. Bond T1** | 2.50 (0.73) | 2.64 (0.92) | -1.660 | .098 | .167 |
| **. Bond T2** | 2.51 (0.80) | 2.62 (0.90) | -1.299 | .195 | .129 |
| **. Concern T1** | 4.01 (0.71) | 3.57 (0.95) | 5.206 | .000^a^ | .518 |
| **. Concern T2** | 3.89 (0.73) | 3.35 (0.96) | 6.173 | .000^a^ | .625 |
| **SOE T1** | 2.00 (0.94) | 2.04 (1.01) | -0.428 | .669 | .041 |
| **SOE T2** | 1.91 (0.93) | 2.28 (1.14) | -3.468 | .001^b^ | .353 |
| **STE T1** | 2.04 (0.98) | 2.25 (0.96) | -2.240 | .026^c^ | .217 |
| **STE T2** | 1.96 (0.90) | 2.37 (1.06) | -4.223 | .000^a^ | .414 |

*Note. n_Spain_* = 179; *n_Chile_* = 224. ^a^*p* ≤ .001; ^b^*p* ≤ .01; ^c^*p* ≤ .05.
