# Supplementary material for: Superordinate identities and self-transcendent emotions: Longitudinal study in Spain and Chile
Source: Front Psychol. 2022 Nov 11;13:989850. doi: 10.3389/fpsyg.2022.989850 (PMC9692013; doi:10.3389/fpsyg.2022.989850)
Supplement: Supplementary file 5 [file Table_5.docx]

***Supplementary Material***

# Supplementary Figures and Tables

**Supplementary Table 5**

*Correlations between Emotional Scales and IWAH Scale by Country and by Time*

| **Spain** | **Community**  **T1** | **Community**  **T2** | **Country**  **T1** | **Country**  **T2** | **Humanity**  **T1** | **Humanity**  **T2** | **Bond**  **T1** | **Bond**  **T2** | **Concern**  **T1** | **Concern**  **T2** |
| --- | --- | --- | --- | --- | --- | --- | --- | --- | --- | --- |
| **SOE T1** | .08 | .03 | .10^d^ | .08 | .13^c^ | .13^c^ | .17^b^ | .20^b^ | .05 | .05 |
| **SOE T2** | -.04 | -.01 | .04 | .10^d^ | .07 | .16^c^ | .12^d^ | .20^b^ | .02 | .07 |
| **STE T1** | .36^a^ | .28^a^ | .34^a^ | .33^a^ | .30^a^ | .28^a^ | .31^a^ | .26^a^ | .19^b^ | .22^b^ |
| **STE T2** | .18^b^ | .11^d^ | .28^a^ | .28^a^ | .27^a^ | .30^a^ | .23^a^ | .30^a^ | .22^b^ | .24^a^ |
| **Chile** |  |  |  |  |  |  |  |  |  |  |
| **SOE T1** | .22^b^ | .23^a^ | .18^b^ | .17^b^ | .23^a^ | .18^b^ | .24^a^ | .23^a^ | .15^c^ | .10^d^ |
| **SOE T2** | .14^c^ | .18^b^ | .16^b^ | .18^b^ | .06 | .16^b^ | .13^c^ | .23^a^ | -.02 | .05 |
| **STE T1** | .27^a^ | .25^a^ | .36^a^ | .32^a^ | .37^a^ | .30^a^ | .36^a^ | .31^a^ | .30^a^ | .26^a^ |
| **STE T2** | .28^a^ | .32^a^ | .29^a^ | .33^a^ | .21^b^ | .31^a^ | .25^a^ | .32^a^ | .12^c^ | .24^a^ |

*Note*: *nSpain* = 179; *nChile* = 224. Pearson correlation (unilateral). ^a^*p* ≤ .001; ^b^*p* ≤ .01; ^c^*p* ≤ .05; ^d^*p* ≤ .10.
