# Supplementary material for: Superordinate identities and self-transcendent emotions: Longitudinal study in Spain and Chile
Source: Front Psychol. 2022 Nov 11;13:989850. doi: 10.3389/fpsyg.2022.989850 (PMC9692013; doi:10.3389/fpsyg.2022.989850)
Supplement: Supplementary file 6 [file Table_6.docx]

***Supplementary Material***

# Supplementary Figures and Tables

**Supplementary Table 6**

*Meta-Analytic Correlations between Emotional Scales and IWAH Scale in the Total Sample*

|  | **Community T1** | **Community T2** | **Country T1** | **Country T2** | **Humanity T1** | **Humanity T2** | **Bond T1** | **Bond T2** | **Concern T1** | **Concern T2** |
| --- | --- | --- | --- | --- | --- | --- | --- | --- | --- | --- |
| **SOE T1** | .161^b^  [0.06, 0.25] | .150^c^  [0.05, 0.24] | .152^c^  [0.06, 0.25] | .137^c^  [0.04, 0.23] | .194^b^  [0.10, 0.29] | .163^b^  [0.07, 0.26] | .221^a^  [0.13, 0.31] | .226^a^  [0.13, 0.32] | .109^c^  [0.01, 0.20] | .082  [-0.02, 0.18] |
| **SOE T2** | .063  [-0.03, 0.16] | .098  [0.00, 0.19] | .107^c^  [0.01, 0.20] | .151^c^  [0.05, 0.24] | .072  [-0.03, 0.17] | .163^c^  [0.07, 0.26] | .130^c^  [0.03, 0.23] | .227^a^  [0.13, 0.32] | -.004  [-0.09, 0.10] | .068  [-0.03, 0.16] |
| **STE T1** | .328 ^a^  [0.24, 0.41] | .274 ^a^  [0.18, 0.36] | .368 ^a^  [0.28, 0.45] | .340 ^a^  [0.18, 0.36] | .363 ^a^  [0.27, 0.44] | .305 ^a^  [0.21, 0.39] | .359 ^a^  [0.27, 0.44] | .300 ^a^  [0.21, 0.39] | .260 ^a^  [0.17, 0.35] | .250 ^a^  [0.16, 0.34] |
| **STE T2** | .248 ^a^  [0.15, 0.34] | .234 ^a^  [0.14, 0.32] | .294 ^a^  [0.20, 0.38] | .323 ^a^  [0.23, 0.41] | .247 ^a^  [0.15, 0.34] | .323 ^a^  [0.23, 0.41] | .252 ^a^  [0.16, 0.34] | .322 ^a^  [0.23, 0.41] | .168 ^c^  [0.07, 0.26] | .251 ^a^  [0.16, 0.34] |

*Note*: Weighted average correlation by sample size and Confidence Interval [95%] (DeCoster & Iselin, 2005). ^a^*p* ≤ .001; ^b^*p* ≤ .01; ^c^*p* ≤ .05.
